# Supplementary material for: Developing a multivariable prediction model to support personalized selection among five major empirically-supported treatments for adult depression. Study protocol of a systematic review and individual participant data network meta-analysis
Source: PLoS One. 2025 Apr 23;20(4):e0322124. doi: 10.1371/journal.pone.0322124 (PMC12017484; doi:10.1371/journal.pone.0322124)
Supplement: S1 Table — PRISMA-P (Preferred Reporting Items for Systematic Review and Meta-Analysis Protocols) 2015 Checklist: Recommended Items to Address in a Systematic Review Protocol. (DOCX) [file pone.0322124.s001.docx]

**S1 Table. PRISMA-P (Preferred Reporting Items for Systematic Review and Meta-Analysis Protocols) 2015 Checklist: Recommended Items to Address in a Systematic Review Protocol.**

| Section and topic | Item No | Checklist item | Page No. |
| --- | --- | --- | --- |
| ADMINISTRATIVE INFORMATION | | |  |
| Title: |  |  |  |
| Identification | 1a | Identify the report as a protocol of a systematic review | 1 |
| Update | 1b | If the protocol is for an update of a previous systematic review, identify as such | NA |
| Registration | 2 | If registered, provide the name of the registry (such as PROSPERO) and registration number | 6 |
| Authors: |  |  |  |
| Contact | 3a | Provide name, institutional affiliation, e-mail address of all protocol authors; provide physical mailing address of corresponding author | 1-2 |
| Contributions | 3b | Describe contributions of protocol authors and identify the guarantor of the review | Author statement |
| Amendments | 4 | If the protocol represents an amendment of a previously completed or published protocol, identify as such and list changes; otherwise, state plan for documenting important protocol amendments | NA |
| Support: |  |  |  |
| Sources | 5a | Indicate sources of financial or other support for the review | Funding statement |
| Sponsor | 5b | Provide name for the review funder and/or sponsor | NA |
| Role of sponsor or funder | 5c | Describe roles of funder(s), sponsor(s), and/or institution(s), if any, in developing the protocol | Funding statement |
| INTRODUCTION | | |  |
| Rationale | 6 | Describe the rationale for the review in the context of what is already known | 5-6 |
| Objectives | 7 | Provide an explicit statement of the question(s) the review will address with reference to participants, interventions, comparators, and outcomes (PICO) | 6 |
| METHODS | | |  |
| Eligibility criteria | 8 | Specify the study characteristics (such as PICO, study design, setting, time frame) and report characteristics (such as years considered, language, publication status) to be used as criteria for eligibility for the review | 6-10 |
| Information sources | 9 | Describe all intended information sources (such as electronic databases, contact with study authors, trial registers or other grey literature sources) with planned dates of coverage | 11 |
| Search strategy | 10 | Present draft of search strategy to be used for at least one electronic database, including planned limits, such that it could be repeated | 11 |
| Study records: |  |  |  |
| Data management | 11a | Describe the mechanism(s) that will be used to manage records and data throughout the review | 11-13 |
| Selection process | 11b | State the process that will be used for selecting studies (such as two independent reviewers) through each phase of the review (that is, screening, eligibility and inclusion in meta-analysis) | 11 |
| Data collection process | 11c | Describe planned method of extracting data from reports (such as piloting forms, done independently, in duplicate), any processes for obtaining and confirming data from investigators | 11-14 |
| Data items | 12 | List and define all variables for which data will be sought (such as PICO items, funding sources), any pre-planned data assumptions and simplifications | 10-11 |
| Outcomes and prioritization | 13 | List and define all outcomes for which data will be sought, including prioritization of main and additional outcomes, with rationale | 10-11 |
| Risk of bias in individual studies | 14 | Describe anticipated methods for assessing risk of bias of individual studies, including whether this will be done at the outcome or study level, or both; state how this information will be used in data synthesis | 13-14,17 |
| Data synthesis | 15a | Describe criteria under which study data will be quantitatively synthesised | 15-16 |
|  | 15b | If data are appropriate for quantitative synthesis, describe planned summary measures, methods of handling data and methods of combining data from studies, including any planned exploration of consistency (such as I^2^, Kendall’s τ) | 15-17 |
|  | 15c | Describe any proposed additional analyses (such as sensitivity or subgroup analyses, meta-regression) | NA |
|  | 15d | If quantitative synthesis is not appropriate, describe the type of summary planned | NA |
| Meta-bias(es) | 16 | Specify any planned assessment of meta-bias(es) (such as publication bias across studies, selective reporting within studies) | 17 |
| Confidence in cumulative evidence | 17 | Describe how the strength of the body of evidence will be assessed (such as GRADE) | 17 |

**Copyright for PRISMA-P (including checklist) is held by the PRISMA-P Group and is distributed under a Creative Commons Attribution Licence 4.0.**

*From: Shamseer L, Moher D, Clarke M, Ghersi D, Liberati A, Petticrew M, Shekelle P, Stewart L, PRISMA-P Group. Preferred reporting items for systematic review and meta-analysis protocols (PRISMA-P) 2015: elaboration and explanation. BMJ. 2015; 349:g7647.*

**S2 Table. TRIPOD-Cluster Checklist of Items to Include When Reporting a Study Developing or Validating a Multivariable Prediction Model Using Clustered Data.**

| **Item No.** | **Description** | **Page No.** |
| --- | --- | --- |
| **Title and abstract** | | |
| 1 | Identify the study as developing and/or validating a multivariable prediction model, the target population, and the outcome to be predicted. | 1 |
| 2 | Provide a summary of research objectives, setting, participants, data source, sample size, predictors,  outcome, statistical analysis, results, and conclusions.* | 3-4 |
| **Introduction** | | |
| 3a | Explain the medical context (including whether diagnostic or prognostic) and rationale for developing or  validating the prediction model, including references to existing models, and the advantages of the study design.* | 5-6 |
| 3b | Specify the objectives, including whether the study describes the development or validation of the  model.* | 5-6 |
| **Methods** | | |
| 4a | Describe eligibility criteria for participants and datasets.* | 7-10 |
| 4b | Describe the origin of the data, and how the data were identified, requested, and collected. | 11-13 |
| 5 | Explain how the sample size was arrived at.* | NA |
| 6a | Define the outcome that is predicted by the model, including how and when assessed.* | 10 |
| 6b | Define all predictors used in developing or validating the model, including how and when measured.* | 10-11 |
| 7a | Describe how the data were prepared for analysis, including any cleaning, harmonisation, linkage, and  quality checks. | 10-11,13-14 |
| 7b | Describe the method for assessing risk of bias and applicability in the individual clusters (eg, using PROBAST). | 17 |
| 7c | For validation, identify any differences in definition and measurement from the development data (eg,  setting, eligibility criteria, outcome, predictors).* | NA |
| 7d | Describe how missing data were handled.* | 14-15 |
| 8a | Describe how predictors were handled in the analyses. | 15-16 |
| 8b | Specify the type of model, all model-building procedures (eg, any predictor selection and penalisation), and method for validation.* | 15-17 |
| 8c | Describe how any heterogeneity across clusters (eg, studies or settings) in model parameter values was  handled. | 15-16 |
| 8d | For validation, describe how the predictions were calculated. | NA |
| 8e | Specify all measures used to assess model performance (eg, calibration, discrimination, and decision  curve analysis) and, if relevant, to compare multiple models. | 16-17 |
| 8f | Describe how any heterogeneity across clusters (eg, studies or settings) in model performance was  handled and quantified. | NA |
| 8g | Describe any model updating (eg, recalibration) arising from the validation, either overall or for particular populations or settings.* | NA |
| 9 | Describe any planned subgroup or sensitivity analysis, (eg, assessing performance according to sources of  bias, participant characteristics, setting). | NA |
| **Results** | | |
| 10a | Describe the number of clusters and participants from data identified through to data analysed. A flow  chart may be helpful.* | NA |
| 10b | Report the characteristics overall and where applicable for each data source or setting, including the key dates, predictors, treatments received, sample size, number of outcome events, follow-up time, and  amount of missing data.* | NA |
| 10c | For validation, show a comparison with the development data of the distribution of important variables  (demographics, predictors, and outcome). | NA |
| 11 | Report the results of the risk of bias assessment in the individual clusters. | NA |
| 12a | Report the results of any across-cluster heterogeneity assessments that led to subsequent actions during  the model’s development (eg, inclusion or exclusion of particular predictors or clusters). | NA |
| 12b | Present the final prediction model (ie, all regression coefficients, and model intercept or baseline estimate  of the outcome at a given time point) and explain how to use it for predictions in new individuals.* | NA |
| 13a | Report performance measures (with uncertainty intervals) for the prediction model, overall and for each  cluster. | NA |
| 13b | Report results of any heterogeneity across clusters in model performance. | NA |
| 14 | Report the results from any model updating (including the updated model equation and subsequent  performance), overall and for each cluster.* | NA |
| 15 | Report results from any subgroup or sensitivity analysis. | NA |
| **Discussion** | | |
| 16a | Give an overall interpretation of the main results, including heterogeneity across clusters in model performance, in the context of the objectives and previous studies.* | NA |
| 16b | For validation, discuss the results with reference to the model performance in the development data, and  in any previous validations. | NA |
| 16c | Discuss the strengths of the study and any limitations (eg, missing or incomplete data, non-  representativeness, data harmonisation problems).* | 18-19 |
| 17 | Discuss the potential use of the model and implications for future research, with a specific view to generalizability and applicability of the model across different settings or (sub)populations.* | 18-19 |
| **Other information** | | |
| 18 | Provide information about the availability of supplementary resources (eg, study protocol, analysis code, datasets).* | Data Availability statement |
| 19 | Give the source of funding and the role of the funders for the present study. | Funding statement |
| PROBAST=prediction model risk-of-bias assessment tool.  *****Item text is an adaptation of one or more existing items from the original TRIPOD (transparent reporting of a multivariable prediction model for individual prognosis or diagnosis) checklist.  *From Debray TPA, Collins GS, Riley RD et al. Transparent reporting of multivariable prediction models developed or validated using clustered data: TRIPOD-Cluster checklist. BMJ 2022;378:e071018; doi:10.1136/bmj- 2022-071018.* | | |
