# Supplementary material for: Developing a multivariable prediction model to support personalized selection among five major empirically-supported treatments for adult depression. Study protocol of a systematic review and individual participant data network meta-analysis
Source: PLoS One. 2025 Apr 23;20(4):e0322124. doi: 10.1371/journal.pone.0322124 (PMC12017484; doi:10.1371/journal.pone.0322124)
Supplement: Attachment — Submitted filename: Response to reviewers - 2024-11-07.docx [file pone.0322124.s003.docx]

**Journal requirements:**

**When submitting your revision, we need you to address these additional requirements.**

**1. Please ensure that your manuscript meets PLOS ONE's style requirements, including those for file naming. The PLOS ONE style templates can be found at** 
[**https://journals.plos.org/plosone/s/file?id=wjVg/PLOSOne_formatting_sample_main_body.pdf**](https://journals.plos.org/plosone/s/file?id=wjVg/PLOSOne_formatting_sample_main_body.pdf) **and** 
[**https://journals.plos.org/plosone/s/file?id=ba62/PLOSOne_formatting_sample_title_authors_affiliations.pdf**](https://journals.plos.org/plosone/s/file?id=ba62/PLOSOne_formatting_sample_title_authors_affiliations.pdf)

Response:

We have double checked the manuscript against the abovementioned templates to ensure it meets PLOS ONE’s style requirements. As a result, we have removed abbreviations in the author affiliations (spelling out full terms instead), adapted heading formats, and updated file names.

**2. We noticed you have some minor occurrence of overlapping text with the following previous publication(s), which needs to be addressed:**

[**https://eprints.whiterose.ac.uk/190928/1/efficacy-and-moderators-of-cognitive-therapy-versus-behavioural-activation-for-adults-with-depression-study-protocol-of-a-systematic-review-and-meta-analysis-of-individual-participant-data.pdf**](https://eprints.whiterose.ac.uk/190928/1/efficacy-and-moderators-of-cognitive-therapy-versus-behavioural-activation-for-adults-with-depression-study-protocol-of-a-systematic-review-and-meta-analysis-of-individual-participant-data.pdf)**?**

**In your revision ensure you cite all your sources (including your own works), and quote or rephrase any duplicated text outside the methods section. Further consideration is dependent on these concerns being addressed.**

Response:

We have compared the manuscript with the abovementioned previous publication by our group. As a consequence, we have added citations in the methods section and rephrased three sentences in the discussion section. In this way, we hope to have addressed all minor text overlap. If, despite our best efforts, any occurrences can still be identified, please let us know which specific sections are of concern and we will be more than happy to address these too.

**3. In the online submission form, you indicated that the collective de-identified individual participant database that will be developed for this study will be available for use by other researchers, provided that the authors of the original studies approve the use of their data for this purpose. Requests can be made with the corresponding author (**[**ellen.driessen@ru.nl**](mailto:ellen.driessen@ru.nl)**). Access (with limited investigator support) will be granted after approval of a study proposal by all authors and a signed data access agreement. 

All PLOS journals now require all data underlying the findings described in their manuscript to be freely available to other researchers, either 1. In a public repository, 2. Within the manuscript itself, or 3. Uploaded as supplementary information.
This policy applies to all data except where public deposition would breach compliance with the protocol approved by your research ethics board. If your data cannot be made publicly available for ethical or legal reasons (e.g., public availability would compromise patient privacy), please explain your reasons on resubmission and your exemption request will be escalated for approval.**

Response:

This manuscript describes the protocol for a study that has not yet been conducted. Therefore, it does not describe any findings. The individual participant database described above has not yet been developed and it is currently not possible to make this available.

We can see how our phrasing in the online submission form might be confusing in this regard and have now adjusted it as follows:

*Data availability is not applicable to this article as no data were analyzed for this study protocol paper.*

**4. As required by our policy on Data Availability, please ensure your manuscript or supplementary information includes the following:**

**A numbered table of all studies identified in the literature search, including those that were excluded from the analyses.**

**For every excluded study, the table should list the reason(s) for exclusion.**

**If any of the included studies are unpublished, include a link (URL) to the primary source or detailed information about how the content can be accessed.**

**A table of all data extracted from the primary research sources for the systematic review and/or meta-analysis. The table must include the following information for each study:**

**Name of data extractors and date of data extraction**

**Confirmation that the study was eligible to be included in the review.**

**All data extracted from each study for the reported systematic review and/or meta-analysis that would be needed to replicate your analyses.**

**If data or supporting information were obtained from another source (e.g. correspondence with the author of the original research article), please provide the source of data and dates on which the data/information were obtained by your research group.**

**If applicable for your analysis, a table showing the completed risk of bias and quality/certainty assessments for each study or outcome.  Please ensure this is provided for each domain or parameter assessed. For example, if you used the Cochrane risk-of-bias tool for randomized trials, provide answers to each of the signalling questions for each study. If you used GRADE to assess certainty of evidence, provide judgements about each of the quality of evidence factor. This should be provided for each outcome.**

**An explanation of how missing data were handled.**

**This information can be included in the main text, supplementary information, or relevant data repository. Please note that providing these underlying data is a requirement for publication in this journal, and if these data are not provided your manuscript might be rejected.**

Response:

This manuscript is a study protocol paper and does not describe any findings. Literature searches, data extraction, and risk of bias assessments have not yet been completed. These will be described in the article reporting the outcomes of the study we describe in the current protocol paper.

**5. Please include captions for your Supporting Information files at the end of your manuscript, and update any in-text citations to match accordingly. Please see our Supporting Information guidelines for more information:** [**http://journals.plos.org/plosone/s/supporting-information**](http://journals.plos.org/plosone/s/supporting-information)**.**

Response:

We now include captions for the Supporting Information files at the end of the manuscript and have updated their in-text citations accordingly.

**Reviewers' comments:

Reviewer's Responses to Questions**

**Comments to the Author

1. Does the manuscript provide a valid rationale for the proposed study, with clearly identified and justified research questions?

The research question outlined is expected to address a valid academic problem or topic and contribute to the base of knowledge in the field.**

**Reviewer #1: Yes**

**2. Is the protocol technically sound and planned in a manner that will lead to a meaningful outcome and allow testing the stated hypotheses?

The manuscript should describe the methods in sufficient detail to prevent undisclosed flexibility in the experimental procedure or analysis pipeline, including sufficient outcome-neutral conditions (e.g. necessary controls, absence of floor or ceiling effects) to test the proposed hypotheses and a statistical power analysis where applicable. As there may be aspects of the methodology and analysis which can only be refined once the work is undertaken, authors should outline potential assumptions and explicitly describe what aspects of the proposed analyses, if any, are exploratory.**

**Reviewer #1: Yes**

**3. Is the methodology feasible and described in sufficient detail to allow the work to be replicable?

Descriptions of methods and materials in the protocol should be reported in sufficient detail for another researcher to reproduce all experiments and analyses. The protocol should describe the appropriate controls, sample size calculations, and replication needed to ensure that the data are robust and reproducible.**

**Reviewer #1: Yes**

**4. Have the authors described where all data underlying the findings will be made available when the study is complete?

The** [**PLOS Data policy**](https://eur01.safelinks.protection.outlook.com/?url=https%3A%2F%2Fc05y1x9s.r.us-east-2.awstrack.me%2FL0%2Fhttps%3A%252F%252Fjournals.plos.org%252Fplosone%252Fs%252Fmaterials-and-software-sharing%2F1%2F010f019232ec1791-78bc9c9b-d535-4a4e-b9ec-6fe3429ff5c3-000000%2F300qHsoVzNFl4Qm0Lnt0yaCIsK4%3D178&data=05%7C02%7CE.Driessen%40psych.ru.nl%7Cd769b2a0519e4d17058c08dcdef2fe58%7C084578d9400d4a5aa7c7e76ca47af400%7C1%7C0%7C638630385563138389%7CUnknown%7CTWFpbGZsb3d8eyJWIjoiMC4wLjAwMDAiLCJQIjoiV2luMzIiLCJBTiI6Ik1haWwiLCJXVCI6Mn0%3D%7C0%7C%7C%7C&sdata=CdD3zApBCs6U4RC9z4MBO6zNCPeLDGMU7HophbuRjSY%3D&reserved=0) **requires authors to make all data underlying the findings described in their manuscript fully available without restriction, with rare exception, at the time of publication. The data should be provided as part of the manuscript or its supporting information, or deposited to a public repository. For example, in addition to summary statistics, the data points behind means, medians and variance measures should be available. If there are restrictions on publicly sharing data—e.g. participant privacy or use of data from a third party—those must be specified.**

**Reviewer #1: Yes**

**5. Is the manuscript presented in an intelligible fashion and written in standard English?

PLOS ONE does not copyedit accepted manuscripts, so the language in submitted articles must be clear, correct, and unambiguous. Any typographical or grammatical errors should be corrected at revision, so please note any specific errors here.**

**Reviewer #1: Yes**

**6. Review Comments to the Author

Please use the space provided to explain your answers to the questions above and, if applicable, provide comments about issues authors must address before this protocol can be accepted for publication. You may also include additional comments for the author, including concerns about research or publication ethics.

You may also provide optional suggestions and comments to authors that they might find helpful in planning their study.

Reviewer #1: Thank you for the opportunity to review this protocol for an outstanding study. This is one of the most well-written protocols and most promising trials I have recently seen. I have only a few minor comments.**

Response:

We thank Reviewer 1 for their thoughtful review and positive feedback. We are very happy to hear that our proposed study is considered promising and of high quality.

**1. There seems to be some confusion regarding who is the corresponding author of this manuscript (ED or ZDC).**

Response:

ED and ZDC share corresponding authorship for this manuscript and are, therefore, both listed as corresponding authors. This is in line with PLOS ONE’s submission guidelines that do “not restrict the number of corresponding authors that may be listed on the article in the event of publication” (<https://journals.plos.org/plosone/s/submission-guidelines>).

**2. The authors may want to include the following reference concerning treatment heterogeneity in the introduction section:
Volkmann C, Volkmann A, Müller CA. On the treatment effect heterogeneity of antidepressants in major depression: A Bayesian meta-analysis and simulation study. Hutson AD, ed. PLoS One. 2020;15(11):e0241497. doi:10.1371/journal.pone.0241497**

Response:

We thank Reviewer 1 for this suggestion and have included the reference [5] in the introduction (page 5, marked up copy) as follows:

*People suffering from depression have a range of therapeutic options, including various antidepressant medications (ADMs) and psychological treatments such as cognitive therapy (CT), behavioral activation (BA), interpersonal psychotherapy (IPT), and short-term psychodynamic psychotherapy (STPP). Although no significant differences in average treatment effects have been found between these interventions [1,2], response is highly heterogeneous [3,4,5] warranting the need for more personalized treatment recommendations [6].*

**3. I have a question regarding the included delivery formats. The manuscript states that guided digital interventions will be included. Are there any restrictions regarding the amount of guidance? This can differ significantly, to the point where human support is reduced to a couple of minutes per patient.**

Response:

This is a good point. To avoid large heterogeneity in the amount of guidance between studies, we decided to exclude guided digital interventions. Our literature searches so far did not identify any study of guided digital interventions meeting the inclusion criteria for this work, suggesting that this decision likely will not lead to a substantial decrease in sample size. We have adjusted the relevant methods section (page 9, marked up copy), which now reads:

*We will include psychotherapies in any delivery format (i.e., face-to-face, telephone, or videoconferencing), as long as a clinician delivers the therapy [30,31]. Bibliotherapy, internet therapy, or other self-help formats will be excluded.*

**4. How will the authors account for the effect of different settings/intensities of care, as both outpatient and inpatient care settings are included?**

Response:

We thank Reviewer 1 for this question. We realize now that inpatient psychiatric care settings by definition do not meet our inclusion criteria, as do partial hospitalization and intensive outpatient programs, since patients in these settings receive more care than psychotherapy or antidepressant monotherapy. Therefore, we have rephrased the relevant section as follows to avoid this confusion (page 9, marked up copy):

*Treatment must not exceed 6 months with no restrictions on the number of sessions [30,31]. Inpatient settings, partial hospitalization programs, and intensive outpatient programs will be excluded, since by definition more care is provided than psychotherapy or antidepressant monotherapy. We will place no other restrictions on the setting in which treatment is delivered (e.g., primary care, outpatient mental health care) [30,31].*

**5. Will there be a minimum depression baseline score for inclusion? How will this be operationalized?**

Response:

As described at page 7 (marked up copy), depression is defined at study-level, as participants 1) meeting specified criteria (e.g., Diagnostic and Statistical Manual of Mental Disorders) for major depressive disorder or another unipolar mood disorder assessed by means of a semi-structured interview or clinicians’ assessment, or 2) presenting a score at or above a validated cut-off indicating the likelihood of clinically significant depressive symptoms on an evaluator-assessed, clinician-assessed, or self-reported measure of depression.

As such, there is no minimum depression baseline score for inclusion. For studies in the first category, baseline depression scores are allowed to vary as long as the participants meet diagnostic criteria for a unipolar mood disorder. For studies in the second category, the minimum depression baseline score is operationalized by the cut-off score of the specific measure used indicating the likelihood of clinically significant depressive symptoms (e.g., Hamilton Depression Rating Scale score ≥ 10; Beck Depression Inventory score ≥ 10).

We have now rephased the relevant section in hopes of providing further clarification:

*Participants will be considered depressed if they meet specified criteria (e.g., Diagnostic and Statistical Manual of Mental Disorders) for major depressive disorder or another unipolar mood disorder assessed by means of a semi-structured interview or clinicians’ assessment, or if they present a score at or above a validated cut-off indicating the likelihood of clinically significant depressive symptoms on an evaluator-assessed, clinician-assessed, or self-reported measure of depression (e.g., Hamilton Depression Rating Scale total score ≥ 10; Beck Depression Inventory total score ≥ 10) [29-31,34].*

**6. Will there be exclusions of participants on an individual patient data level if the authors find indications that some inclusion criteria are not met by single persons in the IPD?**

Response:

Yes, it is part of our data integrity check procedure to check for participants reporting values that are in conflict with the eligibility criteria of the study in which they were enrolled or the meta-analysis proposed (e.g., age <18). We considered this included in our checks “for inconsistent, invalid, or out-of-range values”, but we now describe this explicitly in the relevant methods section (page 14, marked up copy) as follows:

*Third, the outcome and predictor variables will be checked for inconsistent, invalid, or out-of-range values [29-31], including values that conflict with the primary study’s or this work’s eligibility criteria (e.g., age < 18). Discrepancies resulting from these data integrity checks will be discussed with the authors [29-31].*

**7. Regarding data checks, it appears that the authors do not compare the means/standard deviations or raw counts with those reported in the publication. This is highly recommended.**

Response:

We agree with Reviewer 1 that it is important to compare the means and standard deviations or raw counts between the publication and the dataset received. This is indeed part of our planned procedures, but we now see how we did not describe it clearly in the manuscript. Therefore, we have rephrased the methods section (page 14, marked up copy) and mention this explicitly:

*We will perform three data integrity checks [29-31]. First, we will check whether the dataset includes the full intention-to-treat sample (defined as all participants randomized to treatment in the study) and otherwise matches the data reported in the published article [29-31]. To this end, means and standard deviations or raw counts for all baseline characteristics, and observed pre-treatment and post-treatment scores reported in the article will also be calculated from the dataset and both will be compared [29-31].*

**8. Multiple imputation of missing moderators (even if they are entirely missing in a study): I am not sufficiently familiar with Network Meta-Analyses to comment in detail, but if feasible, I recommend performing a sensitivity analysis where imputation is conducted within each study only.**

Response:

We thank Reviewer 1 for this suggestion. Indeed, performing the imputation separately within each study could be useful as a sensitivity analysis or as the primary analysis. Results should not materially change; and if they do, we should place less trust in them. At the same time, when there are systematically missing variables, a separate imputation in each study is not possible. If, for example, in a study there was no information collected at all about marital status, there is no way to meaningfully impute this variable using information from this study alone. In this case, we are forced to use an imputation method that borrows information from other studies that did assess this variable. Of note, the methods for imputation we describe do take into account the stratification of patients in different studies, so that even though each study is not analyzed separately, the information about study allocation is preserved.

We now incorporate this in the section on missing data imputation (page 15, marked up copy):

*Data will be imputed for variables that are ‘systematically’ missing for certain studies and values that are ‘sporadically’ missing for certain individual participants. We will use methods for multiple imputation that account for the clustering of participants in different studies [58,59], utilizing both baseline variables as well as all intermediate and endpoint outcomes available in the datasets [60]. If there are no systematically missing predictors in the studies, we will perform multiple imputation of missing data for each study separately as our primary analysis, and use the procedure described above as sensitivity analysis. Single imputation will also be considered, for instance for imputation of baseline variables, if it follows recommended advice [61].*

**7. PLOS authors have the option to publish the peer review history of their article (**[**what does this mean?**](https://eur01.safelinks.protection.outlook.com/?url=https%3A%2F%2Fc05y1x9s.r.us-east-2.awstrack.me%2FL0%2Fhttps%3A%252F%252Fjournals.plos.org%252Fplosone%252Fs%252Feditorial-and-peer-review-process%2523loc-peer-review-history%2F1%2F010f019232ec1791-78bc9c9b-d535-4a4e-b9ec-6fe3429ff5c3-000000%2FED2_UQbxdHLPn9Zh2YgkhxzGNyc%3D178&data=05%7C02%7CE.Driessen%40psych.ru.nl%7Cd769b2a0519e4d17058c08dcdef2fe58%7C084578d9400d4a5aa7c7e76ca47af400%7C1%7C0%7C638630385563153369%7CUnknown%7CTWFpbGZsb3d8eyJWIjoiMC4wLjAwMDAiLCJQIjoiV2luMzIiLCJBTiI6Ik1haWwiLCJXVCI6Mn0%3D%7C0%7C%7C%7C&sdata=gbjLroBqHZODNbkSLE%2Bd12GD3XOywBKq1OL9iE4%2BoDQ%3D&reserved=0)**). If published, this will include your full peer review and any attached files.

If you choose “no”, your identity will remain anonymous but your review may still be made public.

Do you want your identity to be public for this peer review? For information about this choice, including consent withdrawal, please see our** [**Privacy Policy**](https://eur01.safelinks.protection.outlook.com/?url=https%3A%2F%2Fc05y1x9s.r.us-east-2.awstrack.me%2FL0%2Fhttps%3A%252F%252Fwww.plos.org%252Fprivacy-policy%2F1%2F010f019232ec1791-78bc9c9b-d535-4a4e-b9ec-6fe3429ff5c3-000000%2FweTVqBtdwOyErVqfkIYe6oN2YGY%3D178&data=05%7C02%7CE.Driessen%40psych.ru.nl%7Cd769b2a0519e4d17058c08dcdef2fe58%7C084578d9400d4a5aa7c7e76ca47af400%7C1%7C0%7C638630385563168924%7CUnknown%7CTWFpbGZsb3d8eyJWIjoiMC4wLjAwMDAiLCJQIjoiV2luMzIiLCJBTiI6Ik1haWwiLCJXVCI6Mn0%3D%7C0%7C%7C%7C&sdata=0mNCLWbCnRIMCU6a1SSvN3l584p8%2Frfriseibq%2FjqRM%3D&reserved=0)**.**

**Reviewer #1: No**

**[NOTE: If reviewer comments were submitted as an attachment file, they will be attached to this email and accessible via the submission site. Please log into your account, locate the manuscript record, and check for the action link "View Attachments". If this link does not appear, there are no attachment files.]

While revising your submission, please upload your figure files to the Preflight Analysis and Conversion Engine (PACE) digital diagnostic tool,** [**https://pacev2.apexcovantage.com/**](https://eur01.safelinks.protection.outlook.com/?url=https%3A%2F%2Fc05y1x9s.r.us-east-2.awstrack.me%2FL0%2Fhttps%3A%252F%252Fpacev2.apexcovantage.com%252F%2F1%2F010f019232ec1791-78bc9c9b-d535-4a4e-b9ec-6fe3429ff5c3-000000%2Fuojte5LFaNGXcsgfcOuk3zOj09U%3D178&data=05%7C02%7CE.Driessen%40psych.ru.nl%7Cd769b2a0519e4d17058c08dcdef2fe58%7C084578d9400d4a5aa7c7e76ca47af400%7C1%7C0%7C638630385563181353%7CUnknown%7CTWFpbGZsb3d8eyJWIjoiMC4wLjAwMDAiLCJQIjoiV2luMzIiLCJBTiI6Ik1haWwiLCJXVCI6Mn0%3D%7C0%7C%7C%7C&sdata=QPxBeaSzXbj7U7EpBhxOyqFnbUWVPkvo%2F1U4ljhkdbk%3D&reserved=0)**. PACE helps ensure that figures meet PLOS requirements. To use PACE, you must first register as a user. Registration is free. Then, login and navigate to the UPLOAD tab, where you will find detailed instructions on how to use the tool. If you encounter any issues or have any questions when using PACE, please email PLOS at** [**figures@plos.org**](mailto:figures@plos.org)**. Please note that Supporting Information files do not need this step.**

Response:

This manuscript does not include any figures.
