# Supplementary material for: Developing a multivariable prediction model to support personalized selection among five major empirically-supported treatments for adult depression. Study protocol of a systematic review and individual participant data network meta-analysis
Source: PLoS One. 2025 Apr 23;20(4):e0322124. doi: 10.1371/journal.pone.0322124 (PMC12017484; doi:10.1371/journal.pone.0322124)
Supplement: Attachment — Submitted filename: Response to reviewers - 2025-03-04.docx [file pone.0322124.s004.docx]

**Journal Requirements:**

**Please review your reference list to ensure that it is complete and correct. If you have cited papers that have been retracted, please include the rationale for doing so in the manuscript text, or remove these references and replace them with relevant current references. Any changes to the reference list should be mentioned in the rebuttal letter that accompanies your revised manuscript. If you need to cite a retracted article, indicate the article’s retracted status in the References list and also include a citation and full reference for the retraction notice.**

Reply
We have double-checked the reference list and can ensure it is complete and correct. We do not cite any papers that have been retracted.

**Reviewers' comments:

Reviewer #1: Thank you for addressing my comments. Most of my concerns have been thoroughly addressed.

However, there is one detail that may require further clarification:

The authors state: "We will include psychotherapies in any delivery format (i.e., face-to-face, telephone, or videoconferencing), as long as a clinician delivers the therapy [30,31]. Bibliotherapy, internet therapy, or other self-help formats will be excluded."

It is somewhat disappointing that, under the current definition, guided and unguided digital interventions are excluded. These are precisely the types of interventions that hold the greatest potential for scaling psychotherapy treatments in the future.

Nevertheless, the authors might want to consider adding a specification to the inclusion/exclusion criteria clarifying how they plan to deal with mixed-methods interventions, including blended treatment formats.

Thank you once again for the opportunity to review this excellent protocol. I am eagerly looking forward to the results of this study.**

Reply
We thank Reviewer 1 for reviewing the revised version of this manuscript. We are very happy to hear that Reviewer 1 considers this protocol of high quality and prior concerns generally addressed.

We acknowledge the potential of digital interventions as scalable depression treatments. Prior work has been conducted aimed at personalizing treatment selection in this area (e.g., <https://pubmed.ncbi.nlm.nih.gov/33471111/>).

We agree that the manuscript would benefit from specifying how mixed-methods interventions and blended treatment formats are dealt with. We propose to exclude such treatment formats too to avoid large heterogeneity in the amount of guidance between studies. Please note that our literature searches so far did not identify any study applying a blended format. As such, we expect this decision will not lead to many studies being excluded from consideration. We have adjusted the relevant methods section (page 9, marked up copy), which now reads:

*We will include psychotherapies in any delivery format (i.e., face-to-face, telephone, or videoconferencing), as long as a clinician delivers the therapy [30,31]. Bibliotherapy, internet therapy, or other self-help formats will be excluded, as will be blended treatment formats that combine clinician-delivered therapy with internet interventions.*
